# Role of MicroRNA-103a Targeting ADAM10 in Abdominal Aortic Aneurysm

**DOI:** 10.1155/2017/9645874

**Published:** 2017-03-05

**Authors:** Tong Jiao, Ye Yao, Bo Zhang, Da-Cheng Hao, Qing-Feng Sun, Jing-Bo Li, Chao Yuan, Bao Jing, Yun-Peng Wang, Hai-Yang Wang

**Affiliations:** ^1^Department of Vascular Surgery, The First Affiliated Hospital of Harbin Medical University, Harbin 150001, China; ^2^Biotechnology Institute, School of Environment and Chemical Engineering, Dalian Jiaotong University, Dalian 116028, China

## Abstract

MicroRNAs (miRNAs) are deregulated in various vascular ailments including abdominal aortic aneurysm (AAA). MiR-103 is involved in vascular, metabolic, and malignant diseases, but whether it participates in the pathogenesis of AAA remains elusive. ADAM10 plays a vital role in the formation of aneurysm, but whether miRs modulate its activity during AAA formation is totally unknown. In this study, we detected the significantly increased protein expression of ADAM10 in angiotensin II induced murine AAA specimens, while the mRNA expression of ADAM10 was similar between AAA and control, suggesting the posttranscriptional regulation. The ADAM10 specific inhibitor GI254023X dramatically reduced the macrophage infiltration of murine abdominal aorta. Bioinformatic predictions suggest that ADAM10 is the target of miR-103a/107 but the binding site is exclusive. At the cellular level, miR-103a-1 suppressed the protein expression of ADAM10, while antisense miR-103a-1 increased its expression. Particularly, with the progression of murine AAA, the mRNA expression of miR-103a/107 substantially decreased and the protein expression of ADAM10 greatly increased. Together, our data afford the new insight that miR-103a inhibited AAA growth via targeting ADAM10, which might be a promising therapeutic strategy to alleviate AAA.

## 1. Introduction

MicroRNAs (miRNAs) are highly conserved, 22-nucleotide-long noncoding RNAs that negatively regulate gene expression at the posttranscriptional level [1]. MiRNA exerts the regulatory activity by interacting with complementary sequences (frequently in the 3′-untranslated region (3′-UTR)) of mRNA targets. These interactions may lead to the protein translation inhibition or target mRNA degradation depending on whether miRNAs and their targets are perfectly complementary. MiRNAs are dysregulated in various vascular diseases and they play an essential role in aneurysm pathogenesis [2, 3]. A number of miRNAs have been inferred to participate in the aneurysm formation [4, 5]. In recent years, miRNAs have been recognized as critical regulators in development and progression of abdominal aortic aneurysm (AAA) [6, 7].

An aortic aneurysm is a balloon-like bulge in the aorta, the large artery that carries blood from the heart through the chest and torso. Aortic aneurysms were the primary cause of 9,863 deaths in 2014 and a contributing cause in more than 17,215 deaths in the United States in 2009 [8]. Although imaging and surgical techniques have substantial progress in the last decades, no effective drug therapy is available until recently [4], and AAA is still life-threatening, especially among elders. It is vital to develop early detection method and efficient drug-based therapies for AAA patients [9, 10].

MiR-103 is a member of the miR-15/107 family [11]. Accumulating data indicate that the deregulation of miR-103 contributes to various diseases, such as myocardial infarction [3], cancer [12], and diabetes [13]. However, whether it is involved in the pathogenesis of AAA has never been reported. The principal TNF-*α* (tumor necrosis factor-*α*) converting enzyme, a disintegrin and metalloproteinase 17 (ADAM17), is involved in the development of human AAA [9]. No study suggests the involvement of some other members of ADAM family, for example, ADAM9 and 12, in the formation of AAA. In recent years, ADAM10 has been found to participate in the development of AAA [14] and thoracic aortic aneurysm [15], but its regulatory factor is elusive. Could ADAM10 be regulated by miRs? Does miR-103 suppress the AAA formation by directly interacting with its targets of aortic tissues? To date, there is no study reporting the implication of miR-103 in the progression of AAA.

In the present study, we determined the potential effect of miR-103 on murine AAA progression. Our data confirmed the direct interaction between miR-103a-3p and ADAM10 and showed that miR-103a-3p has lower expression level in AAA samples, accompanied by the increased expression of ADAM10 proteins. We found that miR-103a, whose expression could be increased by its SNP, inhibited ADAM10 protein expression in vitro. Given that the ADAM10 inhibitor reduced the inflammation of murine aortic wall and the formation of AAA and enhanced expression of miR-103a and/or interfering with ADAM10 function might be a promising AAA therapeutic strategy.

## 2. Materials and Methods

### 2.1. Angiotensin (Ang) II Infusion Model and Murine Samples

Osmotic pumps (Alzet model 2004) containing Ang II (1 *μ*g/kg per minute; Sigma-Aldrich) were implanted in 10-week-old apoE−/− male mice (day 0; C57BL/6J background). The aorta diameter was determined by ultrasonic imaging [7].

AAA samples and their adjacent normal tissues were collected from AAA mouse in the First Affiliated Hospital of Harbin Medical University and immediately stored in liquid nitrogen until use. No mice had been treated with radiotherapy or chemotherapy before surgery. The study was approved by the Ethics Committee of the 1st Affiliated Hospital of Harbin Medical University.

### 2.2. Cell Culture

Smooth muscle cells (SMCs) were grown in RPMI1640 medium (GIBCO Laboratories, NY, US) and HEK293T cells were grown in DMEM medium. All media were supplemented with 10% fetal bovine serum (Sigma), 100 U/mL penicillin G, and 100 *μ*g/mL streptomycin (GIBCO). All cells were cultured at 37°C in a humidified incubator containing 5% CO_2_.

### 2.3. Vectors Construction, Oligonucleotide Synthesis, and Transfection

Table 1 listed all related DNA sequences. Cells were plated at 60% to 70% confluency on the day before transfection. Human microRNA mimics and the corresponding antisense RNAs were synthesized by Takara (Dalian, China). The microRNAs and antisense RNAs were transfected at a final concentration of 20 nmol/L, using lipofectamine 2000 (Invitrogen) according to the manufacturer's recommendations.

### 2.4. Quantitative Real-Time PCR (qRT-PCR)

Quantitative real-time RT-PCR was conducted similar to that described previously [9, 16, 17]. Total RNA, including ≥18 nt miRs, was isolated using miRNeasy Mini Kit (Qiagen, Germany) according to the manufacturer's instructions. Afterwards, RNA was reverse transcribed, using ReverTra Ace qPCR RT Master Mix with gDNA Remover (Toyobo, Shanghai; for ADAM10) and miScript II RT Kit (Qiagen; for miRs), respectively. Subsequent qRT-PCR was performed using SYBR Green Real-Time PCR MasterMix-plus (Toyobo), miScript SYBR Green PCR Kit, and miScript Primer Assay (Qiagen), on a CFX96 Real-Time PCR System (Bio-Rad, US) as follows: 95°C for 30 s and then 40 cycles of 95°C for 5 s and 60°C for 30 s and one step of 95°C for 15 s. Each amplification reaction was performed in triplicate in a final volume of 20 *μ*L containing 2 *μ*L of the cDNA. Fluorescence was detected at the end of each cycle. The expression levels of miRNAs were normalized to U6 and were calculated using the 2^−ΔΔCt^ method.

### 2.5. Western Blot Analysis and Immunohistochemistry (IHC)

To determine the level of ADAM10 and other protein expressions, total cell or tissue extracts were extracted in cell lysis buffer (Millipore), followed by SDS-PAGE and Western blot analysis. Equal quantities of tissue lysates were resolved by SDS-PAGE, transferred onto PVDF membranes (Roche Diagnostics, Indianapolis, IN, USA) using electroblotting, probed with specific primary antibodies, followed by horseradish peroxidase- (HRP-) conjugated secondary antibodies, and then analyzed. The primary antibodies used were rabbit polyclonal to ADAM10 ADAM10 (1 : 1,000; Abcam, ab1997, Cambridge, UK), rabbit polyclonal to ADAM17 (Abcam, ab2051), rabbit polyclonal to ADAM9 (Abcam, ab186833), rabbit polyclonal to ADAM12 (Abcam, ab39155), and monoclonal rabbit anti-human GAPDH antibody (1 : 500; Santa Cruz Biotechnology, Inc., CA, USA) and were detected using HRP-conjugated goat anti-rabbit IgG antibody (1 : 500; Millipore) in blocking solution. The 75 kDa band was used to analyze changes in ADAM10 expression in all the experiments. Immunoreactive protein bands were visualized using a Luminata Crescendo Western HRP Substrate (Millipore), scanned, and quantified for pixel density by the optical density (OD) method, using the Tanon 5200 automatic chemiluminescence imaging system (Shanghai, China).

For detecting CX3CL1, TNF-*α*, IL-6R, and VE-cadherin in aorta tissues and cultured cells, the rabbit polyclonal to CX3CL1 (Abcam, ab25088), rabbit polyclonal to TNF-*α* antibodies (Abcam, ab9739), rat monoclonal to IL6R (Abcam, ab83053), and rabbit monoclonal to VE-cadherin (Abcam, ab205336) were used as the primary antibodies, followed by the procedures as described above.

Immunohistochemical analysis was performed to determine ADAM10 protein localization and expression. The primary antibody (rabbit anti-human ADAM10; Abcam) was diluted at 1 : 500 and ADAM10 binding was visualized using the standard avidin/biotinylated enzyme complex-HRP staining procedure, with 3,3′-diaminobenzidine as a chromogen and a HRP-conjugated goat anti-rabbit IgG antibody (1 : 500; Millipore). The sections were examined using an optical microscope (BX40; Olympus Corporation, Tokyo, Japan). Cells that contained brown-yellow stained granules in the membrane and cytoplasm were considered positive. Next, five hundred cells in five randomly selected fields under high magnification were counted. Presence of <5% of positive cells was classified as (−), then 5–50% as (+), 50–75% as (++), and >75% as (+++).

For detecting Mac-2 in aorta tissues, the purified monoclonal anti-mouse Mac-2 antibody (BioLegend, US) was used, followed by the procedures as described above.

### 2.6. Luciferase Reporter Assay

To construct the psiCHECK2-ADAM10-3′UTR plasmid that contained the potential binding sites of the ADAM10 3′-UTR downstream of the firefly luciferase gene, a 2346 bp sequence was amplified and inserted into the XhoI and NotI sites of the psiCHECK-2 Luciferase vector (Promega, Madison, WI, USA). The plasmid with the ADAM10 3′-UTR, where the miR-103 target site was subjected to the site-directed mutagenesis, was also constructed. Site-directed Gene Mutagenesis Kit was used for site-directed mutagenesis of ADMA10 mutated clone (Beyotime NO.D0206). HEK293T cells were used to measure luciferase activity. When they grew to 60–70% confluence, cells were cotransfected with 100 ng luciferase plasmid and 50 ng Renilla luciferase plasmid (Ambion, Austin, TX, USA) along with 650 ng miR-103 mimic or NC as described above. After incubation for 48 h at 37°C, the luciferase activity was quantified with the Dual-Luciferase Reporter Assay System (Promega). The Renilla luciferase activity was used as internal control and the firefly luciferase activity was calculated as the mean ± SD after being normalized by Renilla luciferase activity.

### 2.7. The Effect of SNP on the Expression Level of miR-103a-1

Search the SNP site of miR-103-1 from Mfolg program version 3.5 (http://mfold.rna.albany.edu/?q=mfold/RNA-Folding-Form) to predict pre-miR-103a-1 rs760057865(C) and pre-miR-103a-1 rs760057865(G) secondary structure. Determine the effect of SNP on miR-103a in vitro cell model, the first, cell transfection. Logarithmic growth phase cells, according to Invitrogen lipofectamine 2000, were transfected into the plasmid and transfection reagent. After 4 to 6 hours, we observed the cell condition that changed the culture medium. After 24 h, we observed the fluorescent, when the rate of fluorescent was more than 80%, took the picture, and than added 500 uL medium. The cells were harvested for RNA isolation. And then we did the Real-Time PCR and collected the cells, total RNAs were extracted with TRIzol, cDNA synthesis was performed using the dNTP, M-MLV-RTase (Promega), and quantitative PCR was performed using the SYBR Master Mixture (Takara). The statistics were analyzed use *F* = 2^−ΔΔCt^.

### 2.8. Bioinformatics and Statistical Analysis

MiRs interacting with ADAM10 3′ UTR were predicted by Targetscan (http://www.targetscan.org), RNAhybrid (http://bibiserv.techfak.uni-bielefeld.de/rnahybrid/), and miRanda (http://www.microrna.org) software.

The software SPSS version 20.0 was used for statistical analysis. All values are expressed as mean ± SEM and all data have been repeated at least three times. Student's *t*-tests were used to determine the statistical significance of differences between groups. Spearman's correlation was applied to identify the correlation between miR-103a expression and ADAM10 expression. Differences with *p* < 0.05 were considered significant.

## 3. Results

### 3.1. ADAM10 Was Upregulated in Murine AAA

**Figure 1:**
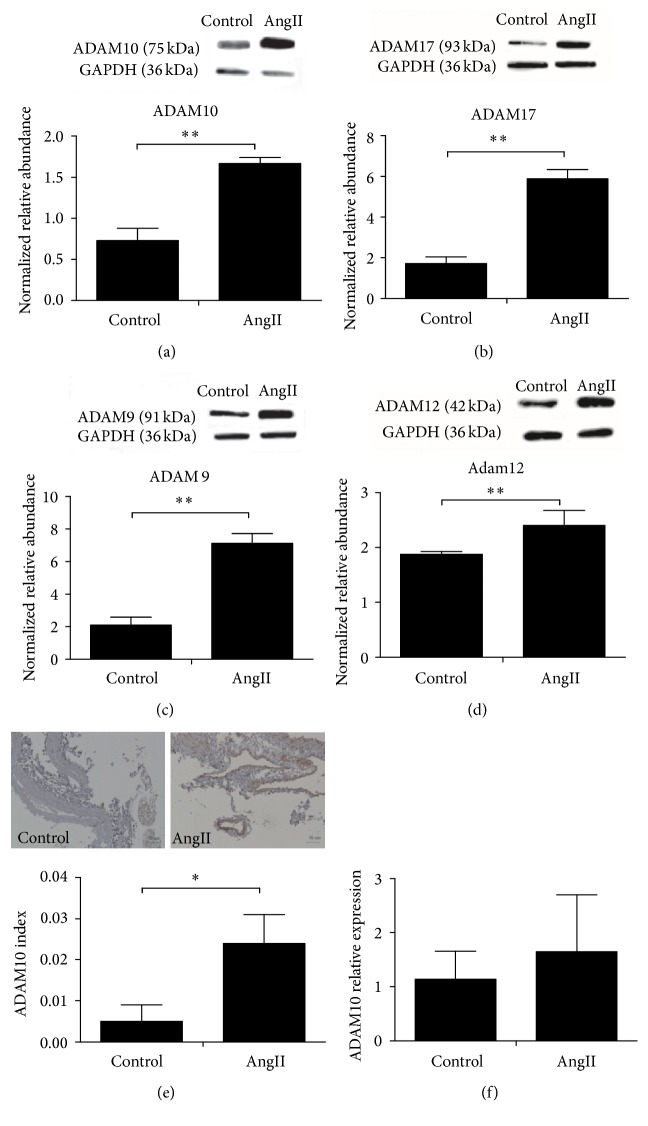
ADAM10 was upregulated in murine AAA. Western blot analysis of (a) ADAM10 (control, 0.73 ± 0.15; AngII, 1.67 ± 0.07;* p*, 0.000); (b) ADAM17 (control, 1.71 ± 0.33; AngII, 5.89 ± 0.45;* p*, 0.000); (c) ADAM9 (control, 2.10 ± 0.49; AngII, 7.12 ± 0.60;* p*, 0.000); and (d) ADAM12 on day 21 of Ang II infusion (control, 1.88 ± 0.05; AngII, 2.40 ± 0.28;* p*, 0.0047). GAPDH is the reference protein. ^*∗*^*p* < 0.05, ^*∗∗*^*p* < 0.01, control group, *n* = 6, and Ang II group, *n* = 6. (e) Examples of IHC staining of ADAM10 in Ang II induced AAA and control aorta (scale bar 10 *μ*m) (control, 0.005 ± 0.004; AngII, 0.024 ± 0.007;* p*, 0.033), control group, *n* = 5, and Ang II group, *n* = 10; (f) ADAM10 gene expression determined by qRT-PCR (control, 1.14 ± 0.52; AngII, 1.65 ± 1.05; *p*, 0.56), control group, *n* = 4, and Ang II group, *n* = 4.

On day 21 of angiotensin (Ang) II infusion, the expression of ADAM10 protein in AAA was more than twice that in control tissues (Figure 1(a)), accompanied by the significantly increased expression of ADAM9, 12, and 17 (*p* < 0.05) (Figures 1(b)–1(d)). Immunohistochemistry (IHC) showed abundant ADAM10 protein (red color) in the aneurysmal aorta (Figure 1(e)). The ADAM10 index of AAA was significantly higher than that of normal aorta (*p* < 0.05). Intriguingly, the mRNA expression of ADAM10 was not different between AAA and control (*p* > 0.05) (Figure 1(f)), implying the posttranscriptional regulation of ADAM10.

### 3.2. ADAM10 Was Involved in the Formation of Murine AAA

**Figure 2:**
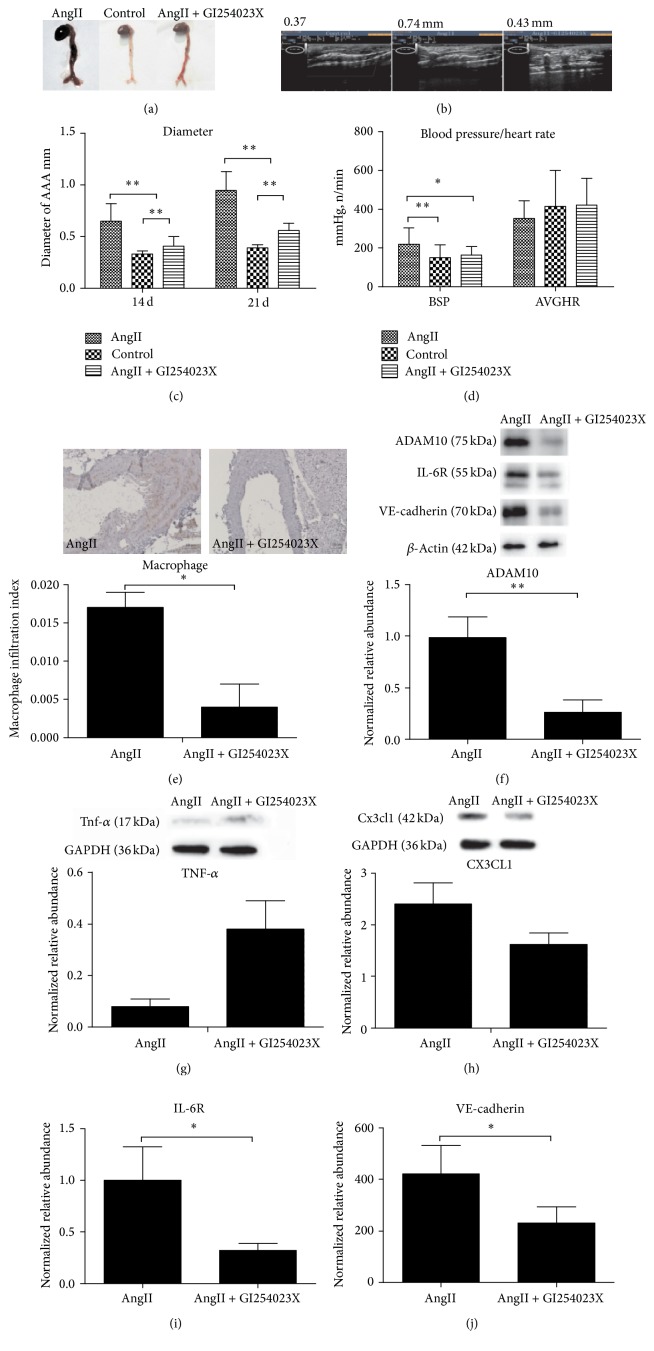
ADAM10 was involved in the formation of murine AAA. (a) Comparison of AAA sizes with/without ADAM10 inhibitor GI254023X by naked eye; (b) the representative ultrasonic images of AAA diameter with/without GI254023X on day 14 of Ang II infusion; (c) comparison of AAA diameters with/without GI254023X by ultrasonic measurement day 14 (AngII, 0.65 ± 0.17; control, 0.33 ± 0.03; AngII + GI254023X 0.41 ± 0.09;* p*, AngII/control, 0.0000; AngII + GI254023X/control, 0.0010; AngII/AngII + GI254023X, 0.0000), Ang II group, *n* = 21, control group, *n* = 3, and AngII + GI254023X group, *n* = 8; day 21 (AngII, 0.95 ± 0.18; control, 0.39 ± 0.03; AngII + GI254023X, 0.56 ± 0.07;* p*, AngII/control, 0.0000; AngII + GI254023X/control, 0.0000; AngII/AngII + GI254023X, 0.0000), Ang II group, *n* = 19, control group, *n* = 3, and AngII + GI254023X group, *n* = 7; (d) comparison of blood pressure and heart rate with/without GI254023X. SBP is systolic blood pressure (AngII, 218.58 ± 83.52; control, 149.21 ± 66.91; AngII + GI254023X, 162.95 ± 43.97;* p*, AngII/control, 0.0030; AngII + GI254023X/control, 0.4585; AngII/AngII + GI254023X, 0.0111), AVG HR is average heart rate (AngII, 351.37 ± 92.53; control, 414.53 ± 183.88; AngII + GI254023X, 420.47 ± 138.96;* p*, AngII/control, 0.1917; AngII + GI254023X/control, 0.8999; AngII/AngII + GI254023X, 0.1023), AngII group, *n* = 19, control group, *n* = 18, and AngII + GI254023X group, *n* = 6; (e) examples of IHC staining of Mac-2 in AAA macrophages with/without GI254023X (scale bar 10 *μ*m) (AngII, 0.017 ± 0.002; AngII + GI254023X, 0.004 ± 0.003;* p*, 0.0151), AngII group, *n* = 4, and AngII + GI254023X group, *n* = 8; (f) comparison of ADAM10 protein expressions with/without GI254023X by Western blot analysis (AngII, 0.98 ± 0.44; AngII + GI254023X, 0.26 ± 0.12;* p*, 0.002), AngII group, *n* = 5, and AngII + GI254023X group, *n* = 4; (g) comparison of TNF-*α* protein expressions with/without GI254023X by Western blot analysis (AngII, 0.08 ± 0.03; AngII + GI254023X, 0.38 ± 0.11;* p*, 0.06), AngII group, *n* = 6, and AngII + GI254023X group, *n* = 6; (h) comparison of CX3CL1 protein expressions with/without GI254023X by Western blot analysis (AngII, 2.40 ± 0.41; AngII + GI254023X, 1.62 ± 0.22;* p*, 0.02), Ang II group, *n* = 6, and AngII + GI254023X group, *n* = 6; (i) comparison of IL-6R protein expressions with/without GI254023X by Western blot analysis (AngII, 0.99 ± 0.33; AngII + GI254023X, 0.32 ± 0.07;* p*, 0.04), AngII group, *n* = 5, and AngII + GI254023X group, *n* = 4; (j) comparison of VE-cadherin protein expressions with/without GI254023X by Western blot analysis (AngII, 421.18 ± 110.00; AngII + GI254023X, 230.48 ± 62.72;* p*, 0.04). GAPDH is the reference protein; ^*∗*^*p* < 0.05, ^*∗∗*^*p* < 0.01, AngII group, *n* = 5, and AngII + GI254023X group, *n* = 4.

GI254023X is a specific inhibitor of ADAM10 [18] and was used here to study the correlation between ADAM10 and murine AAA. On day 14 of angiotensin II infusion, the administration of GI254023X significantly reduced the size of the AAA (Figure 2(a)), and the aorta diameter was decreased from 0.74 mm (no inhibitor) to 0.41 mm (Figures 2(b) and 2(c)), which was comparable to that of control (0.37 mm) (esote MyLab-60, probe 6–18 hz). On day 21, the inhibitory effects of GI254023X were still significant. The MSBP (mean systolic blood pressure) was similar between control group and GI254023X group (Figure 2(d)), which was substantially lower than that of angiotensin II group.

Correspondingly, GI254023X noticeably inhibited the macrophage infiltration within the murine aortic walls, which is supported by the reduced Mac-2 protein (red color) [19] of the macrophage surface in IHC (Figure 2(e)). Western blot analysis confirmed that the ADAM10 protein level in AngII group was nearly four times that in GI254023X group (Figure 2(f)). Moreover, GI254023X suppressed the protein expression of (C-X3-C motif) ligand 1 (CX3CL1) (Figure 2(h)), IL-6R (Figure 2(i)), and VE-cadherin (Figure 2(j)), an endothelial proinflammatory chemokine that binds monocyte CX3C chemokine receptor 1 (CX3CR1) [20, 21], while it did not decrease the expression of TNF-*α* (Figure 2(g)), a substrate of ADAM17. These data suggest that ADAM10 could enhance the macrophage infiltration, exacerbate the inflammatory response, and promote the AAA formation in mice.

### 3.3. ADAM10 Is the Direct Target of miR-103a/107

**Figure 3:**
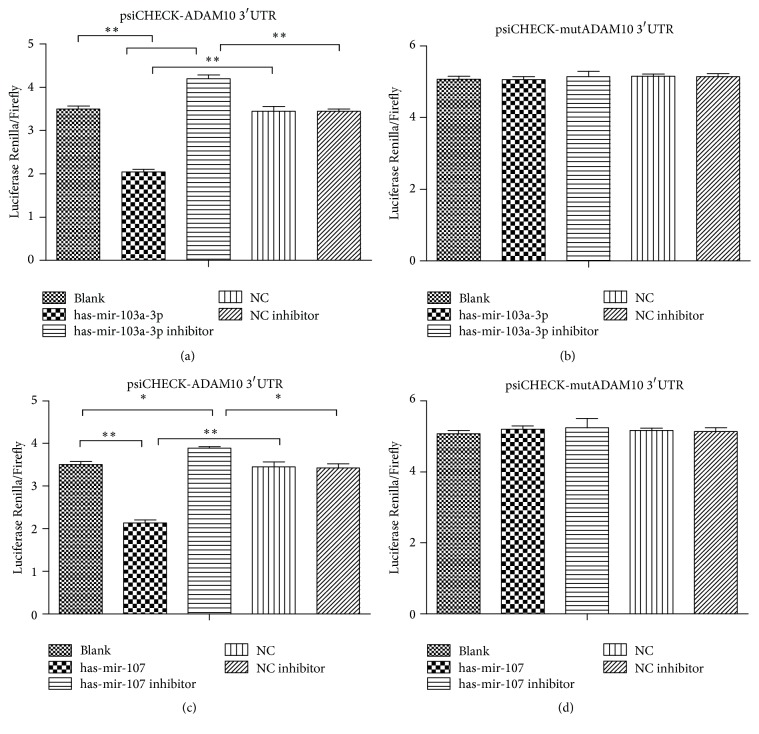
The dual-luciferase reporter assay indicates that ADAM10 is the direct target of miR-103a/107. (a) Luciferase activities on the cotransfection of the reporter plasmid with normal ADAM10 3′UTR (blank, 3.503 ± 0.0697; hsa-mir-103a-3p, 2.048 ± 0.0587; hsa-mir-103a-3p inhibitor, 4.196 ± 0.0924; NC, 3.453 ± 0.1123; NC inhibitor, 3.453 ± 0.0513;* p*, 3′UTR ADAM10 + hsa-mir-103a-3p/3′UTR ADAM10, 0.000155; 3′UTR ADAM10 + hsa-mir-103a-3p/3′UTR ADAM10 + NC, 0.004107; 3′UTR ADAM10 + hsa-mir-103a-3p inhibitor/3′UTR ADAM10, 0.002351; 3′UTR ADAM10 + hsa-mir-103a-3p inhibitor/3′UTR ADAM10 + NC inhibitor, 0.011695); (b) the effect of mutated ADAM10 3′UTR on the luciferase activity when cotransfecting blank, human miR-103a-3p, 103a inhibitor, NC, or NC inhibitor (blank, 5.071 ± 0.0938; hsa-mir-103a-3p, 5.061 ± 0.0910; hsa-mir-103a-3p inhibitor, 5.146 ± 0.1471; NC, 5.164 ± 0.0577; NC inhibitor, 5.135 ± 0.0961;* p*, 3′UTR mut ADAM10 + hsa-mir-103a-3p/3′UTR mut ADAM10, 0.933656; 3′UTR mut ADAM10 + hsa-mir-103a-3p/3′UTR mut ADAM10 + NC, 0.346206; 3′UTR mut ADAM10 + hsa-mir-103a-3p inhibitor/3′UTR mut ADAM10, 0.177698; 3′UTR mut ADAM10 + hsa-mir-103a-3p inhibitor/3′UTR mut ADAM10 + NC inhibitor, 0.811131); (c) luciferase activities on the cotransfection of the reporter plasmid with normal ADAM10 3′UTR (blank, 3.503 ± 0.0697; hsa-mir-107, 2.139 ± 0.0671; hsa-mir-107 inhibitor, 3.892 ± 0.0292; NC, 3.453 ± 0.1123; NC inhibitor, 3.425 ± 0.0982;* p*, 3′UTR ADAM10 + hsa-mir-107/3′UTR ADAM10, 0.001753; 3′UTR ADAM10 + hsa-mir-107/3′UTR ADAM10 + NC, 0.000443; 3′UTR ADAM10 + hsa-mir-107 inhibitor/3′UTR ADAM10, 0.020667; 3′UTR ADAM10 + hsa-mir-107 inhibitor/3′UTR ADAM10 + NC inhibitor, 0.013883); (d) the effect of mutated ADAM10 3′UTR on the luciferase activity when cotransfecting blank, human miR-107, 107 inhibitor, NC, or NC inhibitor (blank, 5.071 ± 0.0938; hsa-mir-107, 5.194 ± 0.0986; hsa-mir-107 inhibitor, 5.233 ± 0.2665; NC, 5.164 ± 0.0577; NC inhibitor, 5.135 ± 0.0961;* p*, 3′UTR mut ADAM10 + hsa-mir-107/3′UTR mut ADAM10, 0.020979; 3′UTR mut ADAM10 + hsa-mir-107/3′UTR mut ADAM10 + NC, 0.544506; 3′UTR mut ADAM10 + hsa-mir-107 inhibitor/3′UTR mut ADAM10, 0.516319; 3′UTR mut ADAM10 + hsa-mir-107 inhibitor/3′UTR mut ADAM10 + NC inhibitor, 0.677649). ^*∗*^*p* < 0.05; ^*∗∗*^*p* < 0.01.

All three software programmes identified targeting sites for miR-103a-3p and miR-107 in the 3′-untranslated region (3′-UTR) of ADAM10 (Fig. S1A–C, in Supplementary Material available online at https://doi.org/10.1155/2017/9645874). The 3′UTR sequence of ADAM10 was amplified from human genomic DNA and cloned into the dual-luciferase reporter vector. The reporter plasmid with the mutated binding site of miR-103a/107 was also constructed. In HEK 293T cells, transfection of human miR-103a-3p led to the significant decrease of luciferase activity (58% of the blank, Figure 3(a); *p* = 0.000155, Figure 3(b)), while transfection of miR-103a inhibitor did not result in the reduction of luciferase activity. Comparing miR-103a group and negative control (NC) group, we found the significantly higher luciferase activity in the latter (*p* = 0.004107), indicating that miR-103a influenced the fluorescence by binding ADAM10 3′UTR. The luciferase activity of 103a inhibitor group was substantially higher than those of blank group and NC inhibitor group (*p* = 0.002351 and 0.011695, resp.), suggesting that the endogenous miR-103a of 293T cells could inhibit the Renilla luciferase of reporter plasmid, and 103a inhibitor suppressed both endogenous and exogenous miR-103a. These data support that miR-103a bound ADAM10 3′UTR, inhibited target gene expression, and suppressed the luciferase activity. Moreover, there could be only one binding site.

In comparison, transfection of either miR103a/inhibitor or NC/inhibitor did not elicit any significant change of luciferase activity (Figures 3(a) and 3(b)), as compared with the blank group. These results suggest that the mutated ADAM10 3′UTR could not bind miR-103a and the latter's inhibitory activity of luciferase was abolished.

As human miR-103a and miR-107, abundantly expressed in vasculature [13, 22] (see below), are evolutionarily closely related and belong to the same miR family [11], we also transfected miR-107 and reporter plasmid into 293T cells and found that the exogenous miR-107 led to the significant decrease of luciferase activity (61% of the blank, Figure 3(c); *p* = 0.001753, Figure 3(d)). The reported activity of miR-107 group was much lower than that of NC group (*p* = 0.000443), indicating that miR-107 influenced the fluorescence by binding ADAM10 3′UTR. The luciferase activity of 107 inhibitor group was substantially higher than those of blank group and NC inhibitor group (*p* = 0.020667 and 0.013883 resp.), suggesting that the endogenous miR-107 of 293T cells could inhibit the Renilla luciferase of reporter plasmid, and 107 inhibitor suppressed both endogenous and exogenous miR-107. In contrast, miR-107 could not bind the mutated ADAM10 3′UTR and its inhibitory effect of luciferase was abolished (Figure 3(d)).

Taken together, these data identified ADAM10 as the direct target of miR-103a/107.

### 3.4. MiR-103a Suppressed the ADAM10 Expression In Vitro

**Figure 4:**
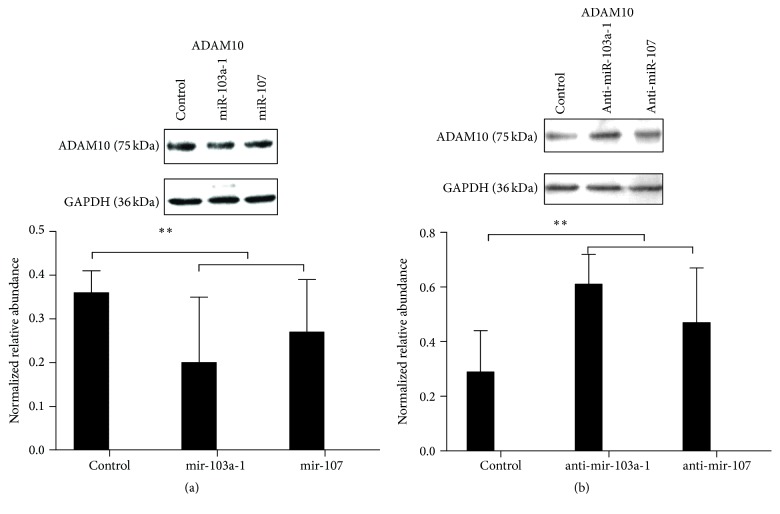
The effects of miR-103a-1, miR-107, and their antisense RNAs on the ADAM10 protein expression. (a) Western blot of control, miR-103a-1, and miR-107 (control, 0.36 ± 0.05; miR-103a-1, 0.20 ± 0.15; miR-107, 0.27 ± 0.12;* p*, miR-103a-1/control, 0.000; miR-107/control, 0.000; miR-107/miR-103a-1, 0.000). (b) Western blot of control and antisense RNAs (control, 0.29 ± 0.15; anti-miR-103a-1, 0.61 ± 0.11; anti-miR-107, 0.47 ± 0.20;* p*, anti-miR-103a-1/control, 0.000; anti-miR-107/control, 0.000; anti-miR-107/anti-miR-103a-1, 0.000). ^*∗∗*^*p* < 0.01.

The overexpression and inhibition of miR-103a in SMCs were performed to determine its role in AAA. The overexpression of miR-103a apparently inhibited the protein expression of ADAM10 in vitro (*p* < 0.05) (Figure 4(a)). In contrast, the overexpression of miR-107 marginally decreased ADAM10 in SMCs. The anti-miR-103a inhibited miR-103a and subsequently significantly upregulated the ADAM10 protein level (*p* < 0.05) (Figure 4(b)). The anti-miR-107 caused the increase of ADAM10 to a lesser degree.

### 3.5. Temporal Expression of ADAM10 and miR-103a/107 in Murine AAA

**Figure 5:**
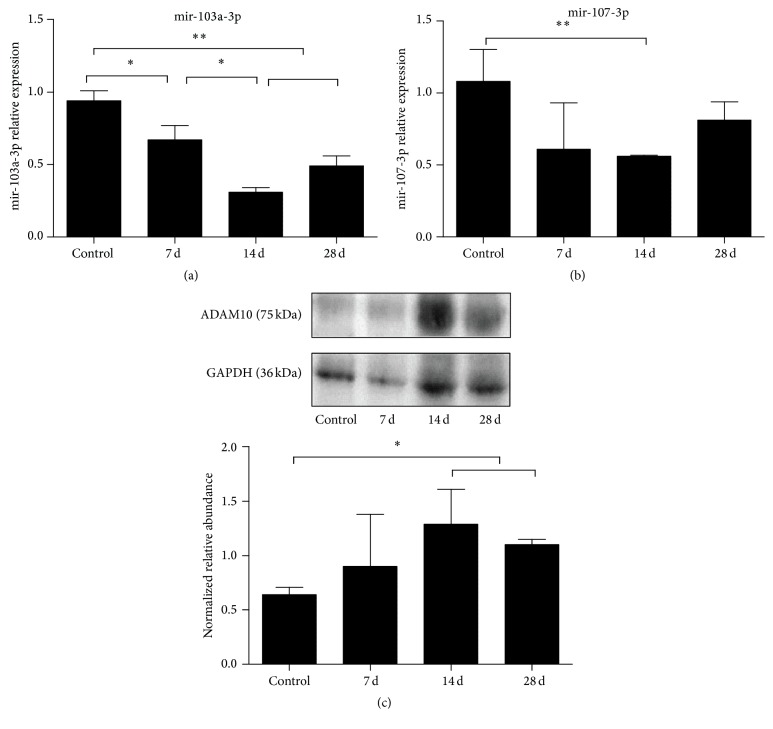
Temporal expression of ADAM10 and miR-103a/107 in AngII induced murine AAA. Control group, *n* = 3; AngII 7 d group, *n* = 6; AngII 14 d group, *n* = 6; AngII 28 d group, *n* = 6. (a), miR103a-3p expression determined by qRT-PCR (control, 0.94 ± 0.07; 7 d, 0.67 ± 0.10; 14 d, 0.31 ± 0.03; 28 d, 0.49 ± 0.07;* p*, 7 d/control, 0.029; 14 d/control, 0.000; 28 d/control, 0.006; 14 d/7 d, 0.014; 28 d/7 d, 0.099; 28 d/14 d, 0.055); (b), miR107-3p expression determined by qRT-PCR (control, 1.08 ± 0.220; 7 d, 0.61 ± 0.32; 14 d, 0.56 ± 0.01; 28 d, 0.81 ± 0.13;* p*, 7 d/control, 0.169; 14 d/control, 0.000; 28 d/control, 0.125; 14 d/7 d, 0.950; 28 d/7 d, 0.293; 28 d/14 d, 0.099); (c) ADAM10 protein expression shown by Western blot analysis (control, 0.64 ± 0.07; 7 d, 0.90 ± 0.48; 14 d, 1.29 ± 0.32; 28 d, 1.10 ± 0.05;* p*, 7 d/control, 0.416; 14 d/control, 0.046; 28 d/control, 0.014; 14 d/7 d, 0.105; 28 d/7 d, 0.528; 28 d/14 d, 0.391). ^*∗*^*p* < 0.05; ^*∗∗*^*p* < 0.01.

We measured miR-103 level, using qRT-PCR, in the murine AAA tissues (Figure 5(a)). MiR-103 was downregulated in AAA and closely associated with AAA formation and progression. We reveal that miR-103 expression was inversely correlated with ADAM10 in murine AAA samples. In aortas of apolipoprotein E-deficient mice on angiotensin II infusion, the mRNA expression of miR103a-3p was significantly decreased on day 14 as compared with control group (*p* < 0.01). MiR103a-3p expression on day 28 was still substantially lower than control (*p* < 0.01), although it was a little higher than that on day 14. The mRNA expression of miR107-3p was also dramatically decreased on day 14 as compared with control (*p* < 0.05) (Figure 5(b)), but its expression on day 28 was not substantially different from that of control. The protein expression of ADAM10 gradually increased between day 0 and day 14 (Figure 5(c)), and the expression level on day 14 was noticeably higher than that of control (*p* < 0.05). Although the ADAM10 expression on day 28 decreased, it was still considerably higher than that of control group (*p* < 0.05). These results suggest the negative modulation of ADAM10 by miR-103a-3p.

### 3.6. The Effect of rs760057865 (C>T) on the Expression Level of miR-103a-1

**Figure 6:**
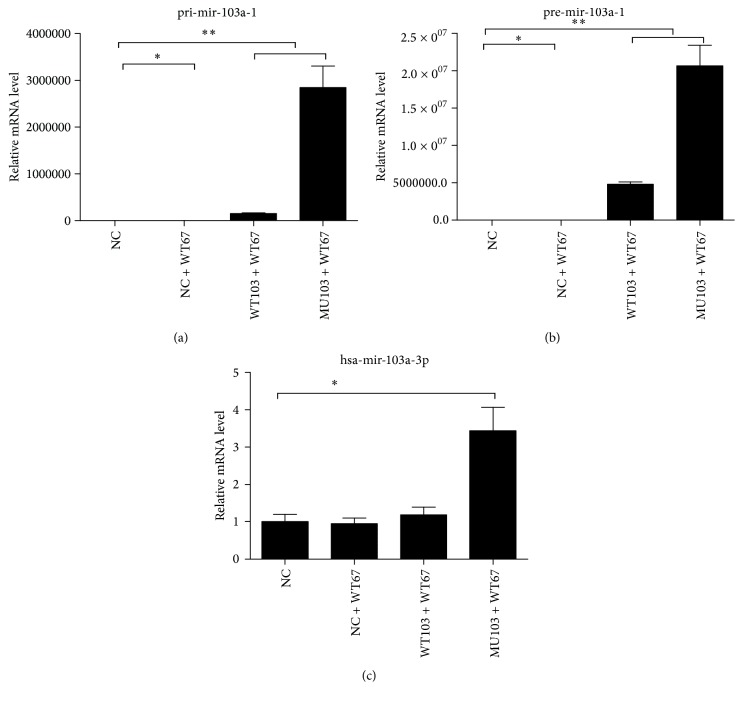
The effect of rs760057865 (C>T) on the expression level of miR-103a-1. MiR-103a-1 and pre-miR-67 were cotransfected into HEK293 cells, and the expression level of the mature miR-67 was used to calibrate the expression level of miR-103a-1. (a) Relative expression level of pri-miR-103a-1 (NC, 1.027 ± 0.302; NC + WT67, 0.454 ± 0.071; WT103 + WT67, 150501.592 ± 17062.329; MU103 + WT67, 2844892.895 ± 460852.778;* p*, NC + WT67/NC, 0.0328; WT103 + WT67/NC, 0.0043; MU103 + WT67/NC, 0.0086). (b) Relative expression level of pre-miR-103a-1 (NC, 1.009 ± 0.166; NC + WT67, 0.508 ± 0.005; WT103 + WT67, 4792513.165 ± 336460.079; MU103 + WT67, 20686901.438 ± 2738072.239;* p*, NC + WT67/NC, 0.0345; WT103 + WT67/NC, 0.0016; MU103 + WT67/NC, 0.0058). (c) Relative expression level of miR-103a (NC, 1.012 ± 0.192; NC + WT67, 0.946 ± 0.157; WT103 + WT67, 1.187 ± 0.208; MU103 + WT67, 3.438 ± 0.623;* p*, NC + WT67/NC, 0.6695; WT103 + WT67/NC, 0.3438; MU103 + WT67/NC, 0.0030). ^*∗*^*p* < 0.05; ^*∗∗*^*p* < 0.01. NC, negative control, empty plasmid; NC + WT67, control; WT67: cel-miR-67-5p mirVana®miRNA mimic (Thermo Fisher number MC22484); WT103 + WT67, wild 103 + 67, WT-Hsa-miR-103a-1; MU103 + WT67, mutation 103 + 67, MT-Hsa-miR-103a-1.

SNP site of miR-103-1 was shown in Fig. S2. In HEK293T cells, the expression level of pri-miR-103a-1 in NC + WT67 was 45.4% of NC group (Figure 6(a)). The expression level of pri-miR-103a-1 in WT103 + WT67 was 150501.5 times that of NC, and MU103 + WT67 expressed much higher level of pri-miR-103a-1 (*p* < 0.01, compared to WT103 + WT67 or NC). The pri-miRis processed to form the precursor microRNA (pre-miR). The expression level of pre-miR-103a-1 in WT103 + WT67 was 4792513.1 times that of NC (Figure 6(b)), and MU103 + WT67 further increased its expression (*p* < 0.01, compared to WT103 + WT67 or NC). Consequently, the transfection of mutant pri-miR-103a-1, containing the SNP rs760057865 (C>T), significantly enhanced the expression of human miR-103a-1 in HEK293T (*p* < 0.01, Figure 6(c)). These results suggest that SNP of the miRNA might influence its expression level, followed by the altered modulation of ADAM10 expression.

## 4. Discussion and Conclusion

Several studies have addressed the involvement of miRs in aneurysm formation and complications [4]. For instance, miR-195 targets a cadre of extracellular matrix proteins and displays significant inverse correlation with aortic diameter [23]. MiR-24 limits aortic vascular inflammation and murine abdominal aneurysm development [7]. Downregulating miR-98 increased the chemotaxis of THP-1 macrophages and MCP-1 induced IL-6 expression in THP-1 cells [6]. Notwithstanding, ADAM family members are not the target of these antianeurysm miRs, and whether miR103a/107 is involved in the pathogenesis of AAA has never been revealed. We thus investigated the mechanisms underlying the role of miR-103 in AAA formation and progression and provide the first batch of evidence that miR-103a/107 may contribute to the pathogenesis of aortic aneurysm via suppressing ADAM10.

ADAMs are a family of transmembrane and secreted metalloendopeptidases. All ADAMs are characterized by a particular domain organization featuring a prodomain, a metalloprotease, a disintegrin, a cysteine-rich, an epidermal-growth factor like, and a transmembrane domain, as well as a C-terminal cytoplasmic tail [24]. Nonetheless, not all human ADAMs have a functional protease domain. Those ADAMs which are active proteases are classified as sheddases because they cut off or shed extracellular portions of transmembrane proteins [25]. Among these ADAMs, ADAM17 was the first sheddase to be identified and plays a crucial role in cell-cell communication, being able to release not only TNF-*α* but also several other transmembrane proteins, including cytokines, adhesion molecules, receptors, and growth factors [8]. ADAM10 is another important membrane-bound sheddase of ADAM family, which is required for the proper functioning of Notch, Eph/ephrin, HER2 receptor, or classic cadherins [24, 26]. During the last decade, ADAM12 emerged as the most strongly functional ADAM in human tumor development. Upregulation of ADAM12 has been described in numerous cancers, including breast, colon, hepatocellular carcinomas, glioblastomas, stomach, oral cavity, esophagus, bladder, lung, and giant cell tumors of bone [27–30]. It has been found that, besides ADAM12, several ADAMs, including ADAM9, 10, 17, and 19, are also involved in the tumorigenesis, invasion, and metastasis of several types of solid tumors [26, 28, 29, 31–35]. In addition, ADAM12 is strongly expressed in the cardiovascular system, erythroid progenitors, brain, and jaw cartilage during zebrafish development [36]. The significant increase of ADAM10 in arteries may contribute to the progression of atherosclerosis [37]. The neutrophils of the intraluminal thrombus exposed to tobacco smoke extract showed increased ADAM10 and 17 content, cleavage of these molecules into active forms, and release of membrane microvesicles carrying mature ADAM10 and detectable ADAM17, which contributed to the damage of the underlying aortic wall [38]. As might be expected, we found that both ADAM10 and ADAM17 proteins were upregulated in the murine AAA in the current study, which is similar to the previous observation that both transmembrane proteases are also elevated in the rat thoracic aortic aneurysm [39]. The data also offer new experimental support for the involvement of ADAM10 in the development of AAA, consistent with previous study [14].

ADAM10 is involved in the shedding of dozens of substrates, such as CX3CL1 [40], IL6-receptor (IL-6R) [40–42], TNF-*α*, and vascular endothelial cadherin (VE-cadherin) [43, 44]. Among these substrates, Notch, CD44, IL-6R, CX3CL1, CXCL16, VEGFR2, and VE-cadherin are of special importance for vascular biology [45]. The cleavage events mediated by ADAM10 play a critical role in various inflammation-related diseases [46], as well as the pathogenesis of vascular diseases [43]. ADAM10 modulates vascular permeability by cleaving endothelial adhesion molecules including VE-cadherin [43, 44]. And it is also the main protease responsible for the ectodomain shedding of various cell surface molecules including the IL-6R and the transmembrane chemokines CX3CL1 and CXCL16 [40, 41]. Interestingly, endogenous IL-6R of both human and murine origin is shed by ADAM17 in an induced manner, whereas constitutive release of endogenous IL-6R is largely mediated by ADAM10 [42]. IL-6R concentrations are typically elevated in a variety of inflammatory disorders [47]. The chemokine CX3CL1, expressed by endothelial cells [21], could interact with the monocyte C-X3-C motif receptor 1 (CX3CR1) and attract monocyte into the inflammatory site of aortic walls. During liver injury, the CX3CL1/CX3CR1 signaling pathway was activated by NF-*κ*B and CD4+, CD8+, and NKT cell infiltration increased in the liver [20]. GI254023X potently blocks the constitutive release of IL-6R, CX3CL1, and CXCL16, which is in line with the reported involvement of ADAM10 but not ADAM17 in this process [41]. Thus, GI254023X may be of use as a preferential inhibitor of constitutive shedding events without effecting the inducible shedding in response to agonists acting [41]. The ADAM10 specific inhibitor GI245023X downregulated the protein expression of CX3CL1, IL-6R, and VE-cadherin, suggesting that, for the first time, ADAM10 could be a key molecule in the formation and progression of murine AAA.

In view of the important role of ADAM10 in the progression of AAA, we further focus on the possible mechanism via ADAM10. ADAM10 is regulated by various miRs in the pathological conditions. For instance, miR-140-5p suppresses tumor cell migration and invasion by targeting ADAM10-mediated Notch1 signaling pathway in hypopharyngeal squamous cell carcinoma [48]. MiR-144 decreased expression of Alzheimer disease-related ADAM10 [49]. ADAM10 is an authentic miR-155 target in the anti-HIV-1 effects of Toll-like receptor 3 stimulation in macrophages [50]. However, the role of ADAM10 and its regulatory miRs in AAA was not well defined, and the interaction between ADAM10 and miR-103a/107 has never been reported for any pathological conditions. We further predicted the interacting miRs of ADAM10 by three stringent software programmes and found that the interaction between ADAM10 and miR103a/107 was direct and the binding site was unique. The temporal expression of miR103a/107 and ADAM10 showed the converse trend, further suggesting that miR103a/107, especially miR103a, could inhibit AAA formation and progression via inhibiting ADAM10.

Of note, GI245023X did not downregulate the protein expression of TNF-*α*. Possible reasons are as follows: first, ADAM17, but not ADAM10, is a known TNF-*α* converting enzyme [9]; second, there might be some unknown regulatory factors of TNF-*α* during AAA development; last but not least, the specific ADAM10 inhibitor GI245023X might not be so specific; that is, it could influence the regulatory factors of TNF-*α*. In this regard, our results highlight the specificity of miR103a/107 in inhibiting ADAM10, which is important in developing a practical therapeutic agent for clinical settings.

SNPs in genes encoding the miRNA sequence may affect miRNA transcription, miRNA processing, and/or the fidelity of the miRNA-mRNA interaction [51]. The functional SNP of miR-200b might be responsible for the susceptibility to large-artery atherosclerotic stroke [52]. The deregulated expression levels of miRs, due to SNPs, have also been observed in various cancers [53]. In the present study, for the first time, we found that a SNP of miR-103a-1 significantly upregulated its expression, which might cause the enhanced suppression of ADAM10 expression and subsequent increased inhibition of AAA. This result implies an alternative strategy with regard to miRNA in AAA diagnosis and therapy. However, before proposing any definitive strategy, more preclinical studies are warranted to elucidate the interrelationship between SNPs of miRNA and AAA.

Therefore, our data collectively demonstrate that miR-103a is an antianeurysm miRNA that could suppress inflammation of the aortic wall and AAA formation and progression by downregulating the protease ADAM10, indicating that miR-103a may represent a new potential diagnostic and therapeutic target for aortic aneurysm. At the cellular level, miR-103a-1 suppressed the in vitro protein expression of ADAM10, while antisense miR-103a-1 increased its expression. For the first time, a SNP of miR-103a was found to augment its expression, implying a novel identification and therapeutic approach.

## Supplementary Material

It was the three software to predict ADAM10's target site of micrna, including TargetScan Human 5.1, miRanda, RNAHybrid.

## Figures and Tables

**Table 1:** Primers for plasmid construction, qRT-PCR, and oligonucleotide.

Primer name		Nucleotide sequence
Plasmid construction		
ADAM10XhoI	F	5′CCGCTCGAG CTGCAGCTTTTGCCTTGGTTCTTC 3′
ADAM10NotI	R	5′ATAAGAATGCGGCCGC TTTTTGGGTGTTTTAAAAAGGTTTATTG 3′
mutADAM10	F	5′CGTGTTCCCTGTTCTTCGTCAACAAATTTTCTTCACTTGCAGGCA 3′
mutADAM10	R	5′TGCCTGCAAGTGAAGAAAATTTGTTGACGAAGAACAGGGAACACG 3′
qPCR		
ADAM10	F	5′CGTGTTCCCTGTTCTTCGTT 3′
ADAM10	R	5′TAGCCTTGATTGGCAGTTGA 3′
miR103a	F	5′AGCAGCAUUGUACAGGGCUAUGA3′
miR107	F	5′AGCAGCAUUGUACAGGGCUAUCA3′
snRNAU6	F	5′GCTTCGGCAGCACATATACTAAAAT3′
snRNAU6	R	5′CGCTTCACGAATTTGCGTGTCAT3′
GAPDH	F	5′TGACTTCAACAGCGACACCCA3′
GAPDH	R	5′CACCCTGTTGCTGTAGCCAAA3′
pri-miR-103a-1	F	5′TGCTCACCACTATACCACCAG3′
pri-miR-103a-1	R	5′CAAAGAATCCCAACTATATGGTC3′
pre-miR-103a-1	F	5′CCCTCGGCTTCTTTACAGTG3′
pre-miR-103a-1	R	5′CAATGCCTTCATAGCCCTG3′
